# Anticonvulsant Activity of Halogen-Substituted Cinnamic Acid Derivatives and Their Effects on Glycosylation of PTZ-Induced Chronic Epilepsy in Mice

**DOI:** 10.3390/molecules23010076

**Published:** 2017-12-29

**Authors:** Ye Cuan, Xirui He, Yuhui Zhao, Jiajun Yang, Yajun Bai, Yin Sun, Qiang Zhang, Zefeng Zhao, Xiaoyang Wei, Xiaohui Zheng

**Affiliations:** 1 Key Laboratory of Resource Biology and Biotechnology in Western China, Ministry of Education, The College of Life Sciences, Northwest University, 229 Taibai Road, Xi’an 710069, China; cuanye2010@163.com (Y.C.); xiruihe6105194@163.com (X.H.); zyhzyh4115@yeah.net (Y.Z.); Yangjiajun0605@163.com (J.Y.); baiyj@nwu.edu.cn (Y.B.); syqsn1108@163.com (Y.S.); ambitionzq@163.com (Q.Z.); zzf598155752@sina.com (Z.Z.); waterxyang@163.com (X.W.); 2 Key Laboratory of Synthetic and Natural Functional Molecule Chemistry of the Ministry of Education, College of Chemistry & Materials Science, Northwest University, Xi’an 710127, China

**Keywords:** epilepsy, cinnamic acid, anticonvulsant activities, glycosylation, neurotoxicity

## Abstract

Epilepsy is a common chronic neurological disorder disease, and there is an urgent need for the development of novel anticonvulsant drugs. In this study, the anticonvulsant activities and neurotoxicity of 12 cinnamic acid derivatives substituted by fluorine, chlorine, bromine, and trifluoromethyl groups were screened by the maximal electroshock seizure (MES) and rotarod tests (Tox). Three of the tested compounds (compounds **3**, **6** and **12**) showed better anticonvulsant effects and lower neurotoxicity. They showed respective median effective dose (ED_50_) of 47.36, 75.72 and 70.65 mg/kg, and median toxic dose (TD_50_) of them was greater than 500 mg/kg, providing better protective indices. Meanwhile, they showed a pentylenetetrazol (PTZ) ED_50_ value of 245.2, >300 and 285.2 mg/kg in mice, respectively. Especially, the most active compound **3** displayed a prominent anticonvulsant profile and had lower toxicity. Therefore, the antiepileptic mechanism of **3** on glycosylation changes in chronic epilepsy in mice was further investigated by using glycomics techniques. Lectin microarrays results showed that epilepsy was closely related to abnormal glycosylation, and **3** could reverse the abnormal glycosylation in *sc*PTZ-induced epilepsy in mice. This work can provide new ideas for future discovery of potential biomarkers for evaluation of antiepileptic drugs based on the precise alterations of glycopatterns in epilepsy.

## 1. Introduction

Mankind has been suffering from epilepsy for thousands of years, and today epilepsy is still one of the most difficult diseases to overcome. Nearly 70 million people in the world suffer from epilepsy, of which thirty percent of patients with epilepsy cannot be cured [1]. Various factors including trauma, infectious diseases, congenital malformations and genetic abnormalities can lead to epilepsy [2]. To date, the antiepileptic drugs is also the most commonly used method for the treatment of epilepsy in clinic, and the commonly used anti-epileptic drugs included phenytoin, carbamazepine, phenobarbital, sodium valproate, etc. [3,4,5]. Some of anti-epileptic drugs greatly alleviate the suffering of many patients with epilepsy, but there are still problems that cannot be ignored. Anti-epileptic drugs have been seriously restricted due to the adverse effects and risks including hepatotoxicity, neurotoxicity, skin lesions, etc. Thus, an urgent need is to develop new anti-epileptic drugs with better safety profiles.

**Figure 1:**
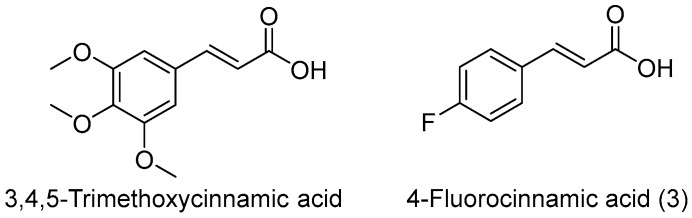
Chemical structure of 3,4,5-trimethoxycinnamic acid and compound **3**.

Traditional Chinese medicine has shown excellent therapeutic potential in many diseases. *Polygala tenuifolia* Willd. has significant sedative effects, and thus has often been used in the treatment of insomnia. 3,4,5-Trimethoxycinnamic acid ((*E*)-3-(3,4,5-Trimethoxyphenyl)acrylic acid, Figure 1), one of the main active ingredients of *P. tenuifolia*, was confirmed to be effective in curing epilepsy [6]. Halogen group elements are common substituent groups, and their unique properties can often improve drug activity. For example, it has been reported that fluorine substitution can have a profound impact on the chemical properties, physical properties, drug disposition (distribution, clearance, route, and metabolism), selective reactivities, and thus effect on the biological activity of drugs [5,6], so one of the methods to improve the biological activity of compounds is by using halogen group elements to replace other substituents in a molecule.

The process of adding sugar chains to proteins and lipids was called glycosylation, which is widely found in various organisms. Glycosylation has become increasingly important in the study of various diseases. In pathological conditions, the abnormal glycosylation of proteins and lipids leads to structural and quantitative changes in the sugar chains of glycosylated proteins and glycolipids, thereby changing their biological functions. In recent years, a large number of studies have reported that abnormal glycosylation is closely related to the development of many diseases such as cancer, and hepatitis [7,8]. Various abnormal glycosylations are strongly associated with the pathogenesis of epilepsy during seizures. Lectin microarrays is a sugar chain detection technique which uses the reactions of various lectins and labeled glycoproteins to detect the sugar chain structure of the sample. Compared with other techniques, lectin microarrays are more simple, rapid and high-throughput for the detection and analysis of glycoproteins. By using lectin microarrays one could further investigate the causes mechanism of epilepsy, and lay the foundation for further epilepsy treatments.

In this study, we screened 12 cinnamic acid derivatives substituted by fluorine, chlorine, bromine, and trifluoromethyl groups for antiepileptic activity and neurotoxicity by using the maximal electroshoch (MES) and rotarod tests, and the results showed the 4-fluorocinnamic acid (**3**, Figure 1), 4-chlorocinnamic acid (**6**) and 4-trifluoromethylcinnamic acid (**12**) showed outstanding anticonvulsant activity compared to the other derivatives. This suggests that halogen-substitution at position 4 of cinnamic acids is beneficial for their anticonvulsant activities. Therefore, we further evaluated theTD_50_ in a rotarod test and ED_50_ values of the three compounds in subcutaneous injection pentylenetetrazol (*sc*PTZ) models in mice. To further investigate the mechanism of the best active compound **3** in the treatment of epilepsy, we established a chronic epilepsy mice model by administering PTZ every other day fifteen times. Then, the glycosylation differences in mice brain was analyzed using lectin microarrays and the results showed that there is abnormal expression of brain protein galactose in *sc*PTZ-induced chronic epilepsy in mice. Through this study, glycomics techniques can be used as a new method in studying glycosylation changes of epilepsy duration and glycopatterns can be used as potential biomarkers for the evaluation of potential antiepileptic drugs.

## 2. Results

### 2.1. Anticonvulsant Activities

According to the data listed in Table 1, all the selected compounds except for bromine-substituted ones displayed anti-convulsant activity. It’s worth noting that different substituent positions can influence the anticonvulsant activity of these compounds. Obviously, the anti-convulsant activity of compound **3**, **6** and **12** was significantly better than that of other ones. Particularly, compound **3** at 100 mg/kg showed a 100% protection rate at 0.5 h in MES test. Moreover, in the MES test compounds **3**, **5**, **10**–**12** displayed prolonged activity as at 2 h.

The active compounds **3**, **6**, **12** were then selected to evaluate the antiepileptic activity in the PTZ-induced seizure model in mice. Every compound was tested at the doses of 100 mg/kg, 200 mg/kg and 300 mg/kg. The results showed that these compounds displayed anticonvulsant activity in *sc*PTZ-induced seizures with a dosage-dependent manner. In particular compound **3** displayed satisfactory activity in this test, and at the highest dose (300 mg/kg) it offered 75% protection against *sc*PTZ-induced tonic seizure and 25% mortality rate according to Table 2. The anticonvulsant activity of compound **6** is similar to compound **3** but with a higher mortality rate at the dose of 300 mg/kg. Compound **12** also dosage-dependently prolonged the latent time, but did not show as good protection against tonic seizure and death percentage than that of compounds **3** and **6**. It can be seen from the above results that compound **3** had a high and promising biological activity.

The excellent potencies of compounds **3**, **6** and **12** in the aforementioned anticonvulsant activity tests led us to further investigate and quantify their pharmacological properties using mice-MES, -*sc*PTZ, and -Tox tests. The results of the ED_50_ and TD_50_ combined with 95% confidence intervals are summarized in Table 3. Analysis of this data revealed that compound **3** was the most promising one, with an ED_50_ value of 47.36 mg/kg for MES and 245.2 mg/kg for the PTZ induced epilepsy test. Additionally, it had significantly better safety profile in the rotarod test (TD_50_ > 500 mg/kg) than the positive reference (STP, TD_50_ < 500 mg/kg).

### 2.2. Lectin Microarrays

**Figure 2:**
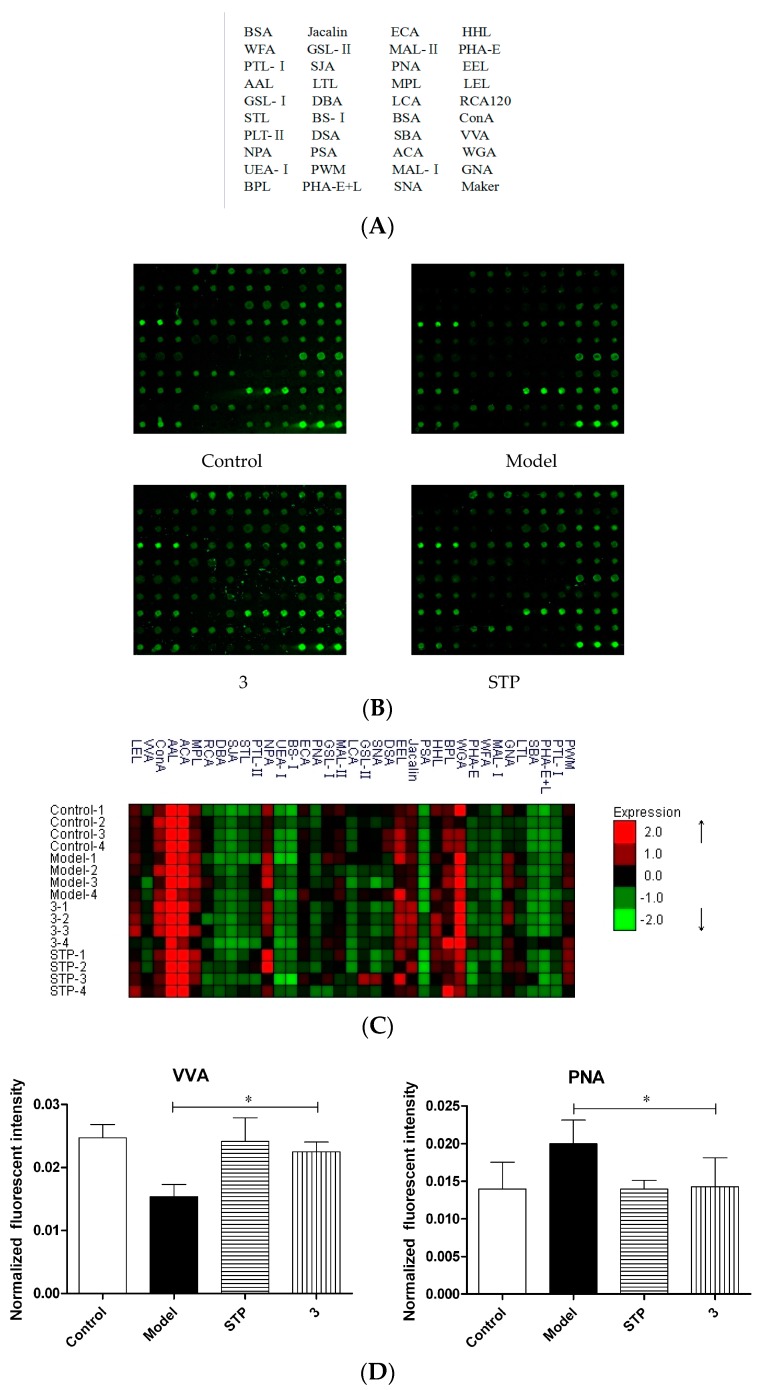
Effect of compound **3** and STP on glycosylation changes in chronic epilepsy in mice by using glycomics techniques. (**A**) the selected 37 lectins; (**B**) the image of the lectin microarrays, which included a total of 16 brains samples; (**C**) Unsupervised average linkage hierarchical cluster analysis of the lectin microarray responses to brain samples. The each brain sample of the control, model, **3** and STP were listed in columns, and the selected 37 lectins were listed in rows. The color and intensity of each square indicated expression levels with respect to the other data in the row. Red, high; black, medium; green, low; (**D**) two lectins (VVA and PNA) revealed differences between control and model group induced by *sc*PTZ. The results are presented as means ± SEM (*n* = 4); * *p* < 0.05, compared with model group.

According to Figure 2, lectin signal patterns were classified into three categories to evaluate whether the glycopatterns of the brain glycoproteins were altered between control, model, STP, and compound **3** groups. Among the 37 lectins, two lectins, peanut agglutinin (PNA) and vicia villosa lectin (VVA) were significantly different in the brains of mice with chronic epilepsy than in the vehicle group. As can be seen from the D diagram, the fluorescence intensity of PNA in the epilepsy model group is stronger than that in the other groups, while the fluorescence intensity of VVA is weaker than the other groups. From these differences, we hypothesized that PTZ induced chronic epilepsy is related to the carbohydrate chains identified by PNA and VVA. It is interesting to note that both compound **3** and STP with good antiepileptic activity can reverse the fluorescence intensity of PNA and VVA to some extent compared with the model group. The sugar chains identified by PNA include Galβ1-3GalNAcα-Ser and Galβ1-3GalNAcα-Thr, which belong to galactose. VVA corresponds to terminal GalNAc, GalNAcα-Ser/Thr(Tn), GalNAcα1-3Gal, which belong to GalNAc. Researchers have found that abnormal glycation is one of the pathological features of early epileptic seizures, and defective galactosylation causes epilepsy [10].

## 3. Discussion

Cinnamic derivatives are aromatic acids which widely distributed in plants such as cinnamon, cereals, legumes, fruits and vegetables [10,11]. Over the past 30 years a large number of studies have found that cinnamic acid and its derivatives have a variety of biological activities including anticancer, anti-inflammatory, anti-hepatotoxicity, antimicrobial, antivirus, etc. [12,13,14,15,16,17]. In Gunia’s study, it was confirmed that the cinnamic acid derivatives have anticonvulsant activity and their study clarifies a new way to discover new potential anticonvulsants [18]. In 2015, a cinnamic acid, 3,4,5-trimethoxycinnamic acid, the main active ingredient isolated from *P. tenuifolia*, has been found to display anticonvulsant effect [19]. Animal models have made great contributions to the treatment of epilepsy. The MES- and PTZ-induced mice models of epilepsy have commonly been used to find new antiepileptic drugs (AEDs) for decades, and many new such drugs that offer appreciable advantages in terms of their favourable pharmacokinetics, improved tolerability, lower toxicity have entered the market [20]. However similar evaluation systems and methods will produce the same therapeutic mechanism to influence the function of AEDs, which has made epilepsy difficult to cure in some patients [21].

Only by using new research methods and techniques can we understand epilepsy more comprehensively. One effective way to study the mechanism of epilepsy is by combining genomics, proteomics, glycomics and other technical advantages. Only through this change can we advance epilepsy research and provide strong support for research and development of new antiepileptic drugs [22,23].

In this study, we demonstrated that halogen-substituted cinnamic acid derivatives (compounds **1**–**12**) have good potential anticonvulsant activities with lower toxicity. The anticonvulsant ability is closely linked to the position of the halogen substituents, and substitutions at the 4 position of the benzene ring were beneficial for antiepileptic activities. Taken altogether, our findings demonstrated the compound **3** represents as new promising candidate with low toxicity. In particular, compound **3** showed marked anticonvulsant activity in MES and PTZ models. MES-induced seizures are especially sensitive to drugs blocking sodium channels, while PTZ-induced seizures are especially sensitive to GABA-mimetic drugs. Based on these models, we speculate that the possibility mechanisms of action of these cinnamic acids are related to sodium channels and/or GABAergic interneurons. In addition, a lectin microarray was used firstly to investigate the glycopatterns in the brains of mice with chronic epilepsy, and systematically compare different or similar alterations of brain glycopatterns between vehicle, model, STP and compound **3**. There were two lectins (PNA and VVA) among the 37 lectins that give significant alterations of brain glycopatterns in control compared with model (all *p* ≤ 0.05). It is very interesting to note that both the STP and compound **3** could reverse this change, which means that both of them prevent the disorders of glycosylation in the brain caused by the PTZ-induced epilepsy model.

In conclusion, in this article we have provided useful information for further research and development of 4-fluorocinnamic acid (**3**). More particularly, we provide insight into the discovery of potential biomarkers for treatment of epilepsy or evaluation of antiepileptic drugs based on the precise alterations of glycopatterns in a PTZ-induced chronic model of epilepsy in mice.

## 4. Materials and Methods

### 4.1. Drugs

All the halogen-substituted cinnamic acids and carbamazepine were purchased from J&K Scientific Ltd. (Beijing, China); PTZ (lot: 10180463) was purchased from Alfa Aesar (Shanghai, China); 3-MP (lot: LD50Q10) and CBZ (lot: LLA0P07) were purchased from J&K Scientific Ltd. (Beijing, China), STP (lot: YDNLC-CP) was from TCI (Shanghai, China).

### 4.2. Animals

Kunming mice of both sexes (25–30 g) were obtained from Xi’an Jiao Tong University Animal Center (Xi’an, China, SCX-2012-003). The mice were kept under well-ventilated conditions at ambient temperature and fed on standard laboratory animal feeds with access to water ad-libitum in Northwest University Center for drug evaluation. All animal experiments were in accordance with and approved by the Animals Ethics Committee of Northwest University (Ethic approval number: NWU-AWC-20170604R).

### 4.3. MES-Induced Seizures in Mice

The methods followed the procedures recommended by the National Institutes of Health (Bethesda, MA, USA) for the development of antiepileptic drugs. Mice were randomly divided into 14 groups of four mice per group. The first group of mice were injected with saline (0.01 mL/g) served as control. Group 2 was received stiripentol (STP) at the dose of 100 mg/kg. Groups 3–14 were received compounds **1**–**12** at the dose of 100 mg/kg through intraperitoneal injection. After the administration of compounds, mice were fixed on the plate, keep ears wet by applying saline, and give the mice an electrical stimulus in 0.5 h, 1 h, 2 h and 3 h after administration. The current, shock duration, frequency and stimulus duration were set and maintained at 50 mA, 60 Hz and 0.25 s, respectively. After electric stimulus, a generalized convulsion is considered not to be protected while lack of generalized convulsion was regarded as protection from seizures.

### 4.4. Rotarod Test

The neurotoxicity of the compounds **1**–**12** were measured in mice using the rotarod test. The mice were trained to place on a diameter 4 cm rod that rotates at 24 rpm. Then, trained mice were selected and randomly divided, and treated with compounds **1**–**12**. 0.5, 1, and 2 h after compound treatment, each mice were placed on rotarod that rotates at 24 rpm. Neurotoxicity is defined as the failure (drop more than 3 times from rotarod in 3 min) of the mice to remain on the rod for 3 min.

### 4.5. PTZ-Induced Acute Seizures in Mice

This method followed the procedures recommended by the National Institutes of Health for the development of antiepileptic drugs. Mice were randomly divided into 11 groups of six mice per group. Group 1 was treated with saline and served as blank control. Group 2 received STP (100 mg/kg) as positive control. Groups 3–11 received compounds **3**, **6** and **12** at the dose of 100 mg/kg, 200 mg/kg and 300 mg/kg respectively. Thirty minutes later, 85 mg/kg of freshly prepared solution of PTZ was administered subcutaneously to all the mice. The mice were observed for 30 min for the onset and incidence of seizures. An episode of clonic spasm with loss of righting reflex for at least 5 s was considered as a convulsion. Lack of threshold convulsion during 30 min of observation was regarded as protection. The number of protected mice was noted and the anticonvulsant properties of the compound were expressed as percentage protection.

### 4.6. PTZ-Induced Chronic Seizures in Mice

Total 24 mice were divided into four groups: control, model, STP and compound **3**, and each group has four animals. All groups of mice except the control group were subcutaneously injected with PTZ (35 mg/kg) every 48 h, 15 times consecutively. Each group was injected with normal saline, compound **3** (100 mg/kg) and STP (100 mg/kg) for half an hour before each injection of PTZ respectively. 30 min after the last treatment, the mice were killed quickly and brain were preserved. Mice were given quantitative food and water during the modeling period.

### 4.7. Brain Processing

The animals’ brains were cut into pieces and transferred to a homogenizer. The precooling tissue lysis buffer was rapidly added to the homogenizer and the mixture was fully ground under ice bath conditions. Then the grinding fluid was transferred to a centrifuge tube (2 mL) and subjected to centrifugation (4 °C, 12,000 rpm, 15 min). After centrifugation, the supernatant, which is the tissue protein extract, was transferred to a new centrifuge tube (1.5 mL). The brain proteins were labeled with Cy3 fluorescent dye (GE Healthcare, Buckinghamshire, UK) and purified using Sephadex G-25 columns according to the instructions provided by the manufacturer. Subsequently, the Cy3-labeled brain proteins were quantified and stored at −20 °C in the dark until use.

### 4.8. Lectin Microarrays

Thirty seven lectins with different binding preferences covering N- and O-linked glycans were spotted on homemade epoxysilane-coated slides. Each lectin was spotted in triplicate per block, with quadruplicate blocks on one slide. After immobilization, the slides were blocked with blocking buffer containing 2% BSA in 1 × PBS (0.01 mol/L phosphate buffer containing 0.15 mol/L NaCl, pH 7.4) for 1 h, rinsed twice with 1 × PBST (0.2% Tween 20 in 1 × PBS) for 5 min each, and finally rinsed in 1 × PBS before drying. The microarrays were scanned using a Genepix 4000B confocal scanner (Axon Instruments, Foster City, CA, USA) set at 70% photomultiplier tube and 100% laser power. The acquired images were analyzed at 532 nm for Cy3 detection by the Genepix 3.0 software (Version 3, Axon Instruments Inc., Union City, CA, USA).

### 4.9. Statistical Analysis

All data were expressed as the mean ± SEM. The statistical analysis was performed using SPSS (Version 20, SPSS Inc., Chicago, IL, USA). Statistical significance was considered at *p* < 0.05. GraphPad Prism 5.0 software (Version 5, GraphPad Software Inc., La Jolla, CA, USA) was been used to correct for multiple comparisons.

## Figures and Tables

**Table 1:**
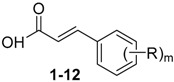
Anticonvulsant Activity and Neurotoxicity of Compounds **1**–**12**: MES and Rotarod Test in Mice *ip* ^a^ (Dose of 100 mg/kg).

Compd.	R	m	Sub. Site	MES ^b^				Tox ^c^		
				0.5 h	1 h	2 h	3 h	0.5 h	1 h	2 h
Saline	---	---	---	0 ^d^/4 ^e^	0/4	0/4	0/4	0 ^f^/4 ^e^	0/4	0/4
STP ^g^	---	---	---	3/4	2/4	1/4	0/4	1/4	0/4	0/4
**1**	F	1	*ortho*	2/4	2/4	0/4	0/4	0/4	0/4	0/4
**2**	F	1	*meta*	3/4	3/4	0/4	0/4	0/4	0/4	0/4
**3**	F	1	*para*	4/4	3/4	1/4	0/4	0/4	0/4	0/4
**4**	Cl	1	*ortho*	2/4	2/4	0/4	0/4	0/4	0/4	0/4
**5**	Cl	1	*meta*	2/4	1/4	0/4	0/4	0/4	0/4	0/4
**6**	Cl	1	*para*	3/4	2/4	1/4	0/4	0/4	0/4	0/4
**7**	Br	1	*ortho*	0/4	0/4	0/4	0/4	0/4	0/4	0/4
**8**	Br	1	*meta*	0/4	0/4	0/4	0/4	0/4	0/4	0/4
**9**	Br	1	*para*	0/4	0/4	0/4	0/4	0/4	0/4	0/4
**10**	CF_3_	1	*ortho*	2/4	1/4	1/4	0/4	0/4	0/4	0/4
**11**	CF_3_	1	*meta*	3/4	2/4	1/4	0/4	0/4	0/4	0/4
**12**	CF_3_	1	*para*	3/4	3/4	1/4	0/4	0/4	0/4	0/4

^a^ The compounds (prepared in 0.5% Tween 80 in 0.9% normal saline) were administered intraperitoneally; ^b^ Maximal electroshock test (the animals were examined at four pretreatment times after drug administration: 0.5, 1, 2 and 3 h); ^c^ Neurological toxicity (determined from the rotarod test at three pretreatment times: 0.5, 1 and 2 h); ^d^ Data indicate number of mice protected; ^e^ number of mice tested; ^f^ the number of mice affected; ^g^ STP = stiripentol.

**Table 2:** Anticonvulsant Activity: *sc*PTZ Test in Mice *ip* ^a^.

Compd.	Dose (mg/kg)	Latent Time (s) ^b^	CSR (%) ^c^	TSR (%) ^c^	MR (%) ^c^
Saline	---	131.1 ± 21.4	100	100	75
STP	100	219.3 ± 23.75 **	100	50	50
**3**	100	140.4 ± 14.5	100	100	75
	200	196.0 ± 58.2 **	100	75	75
	300	280.5 ± 59.2 **	100	25	25
**6**	100	128.6 ± 17.3	100	100	75
	300	160.5 ± 54.4 *	100	50	75
	300	214.0 ± 44.7 **	100	25	50
**12**	100	145.2 ± 18.8	100	100	75
	200	181.0 ± 43.4 *	100	75	75
	300	206 ± 49.0 **	100	25	75

^a^ The selected compounds (prepared in 0.5% Tween-80 in 0.9% normal saline) were administered intraperitoneally; ^b^ Subcutaneous injection PTZ (85 mg/kg, prepared in 0.9% normal saline) at 0.5 h after drug administration; ^c^ The CSR (clonic seizures rate), TSR (tonic seizure rate) and MR (mortality rate) induced by subcutaneous injection PTZ at pretreatment times 0.5 h. The results are presented as means ± SEM (*n* = 4), * *p* < 0.05, ** *p* < 0.01 compared with saline.

**Table 3:** Quantitative Anticonvulsant Data in Mice *ip* ^a^ (ED_50_, TD_50_).

Compd.	TPE (h) ^b^	MES, ED_50_ (mg/kg)	PTZ, ED_50_ (mg/kg)	Tox, TD_50_ (mg/kg)
STP	---	240 [9]	115 [9]	<500
3	0.5	47.36 (17.08–67.95)	245.2	>500
6	0.5	75.72 (56.85–104.67)	>300	>500
12	0.5	70.65 (50.74–121.59)	285.2	>500

^a^ The compounds were administered intraperitoneally to adult male KM mice. ED_50_ and TD_50_ values are in mg/kg; ^b^ Subcutaneous injection PTZ (85 mg/kg, prepared in 0.9% normal saline) at 0.5 h after drug administration. Numbers in parentheses are 95% confidence intervals determined by Probit analysis.
